# Supplementary figures and images for: A TSHZ3 Frame-Shift Variant Causes Neurodevelopmental and Renal Disorder Consistent with Previously Described Proximal Chromosome 19q13.11 Deletion Syndrome
Source: Genes (Basel). 2022 Nov 23;13(12):2191. doi: 10.3390/genes13122191 (PMC9778592; doi:10.3390/genes13122191)

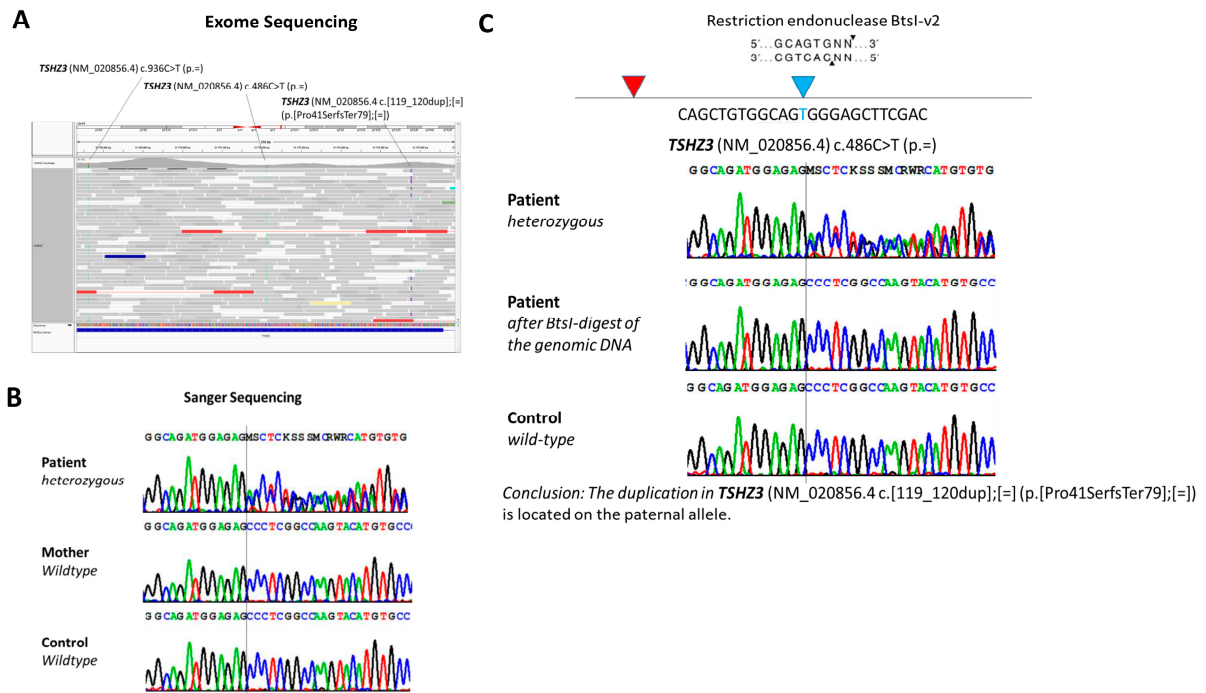

Supplementary Figure S1

Supplement: Supplementary file 1 [file genes-13-02191-s001.zip › genes-1978389-supplementary.pdf]
